# Possible Implications of Bacteriospermia on the Sperm Quality, Oxidative Characteristics, and Seminal Cytokine Network in Normozoospermic Men

**DOI:** 10.3390/ijms23158678

**Published:** 2022-08-04

**Authors:** Eva Tvrdá, Daniel Lovíšek, Eliška Gálová, Marianna Schwarzová, Eva Kováčiková, Simona Kunová, Jana Žiarovská, Miroslava Kačániová

**Affiliations:** 1 Institute of Applied Biology, Faculty of Biotechnology and Food Sciences, Slovak University of Agriculture, Tr. A. Hlinku 2, 949 76 Nitra, Slovakia; 2 Institute of Biotechnology, Faculty of Biotechnology and Food Sciences, Slovak University of Agriculture, Tr. A. Hlinku 2, 949 76 Nitra, Slovakia; 3 Department of Genetics, Faculty of Natural Sciences, Comenius University, Ilkovičova 6, Mlynská Dolina, 842 15 Bratislava, Slovakia; 4 Institute of Nutrition and Genomics, Faculty of Agrobiology and Food Resources, Slovak University of Agriculture, Tr. A. Hlinku 2, 949 76 Nitra, Slovakia; 5 AgroBioTech Research Centre, Slovak University of Agriculture, Tr. A. Hlinku 2, 949 76 Nitra, Slovakia; 6 Institute of Food Sciences, Faculty of Biotechnology and Food Sciences, Slovak University of Agriculture, Tr. A. Hlinku 2, 949 76 Nitra, Slovakia; 7 Institute of Plant and Environmental Sciences, Faculty of Agrobiology and Food Resources, Slovak University of Agriculture, Tr. A. Hlinku 2, 949 76 Nitra, Slovakia; 8 Institute of Horticulture, Faculty of Horticulture and Landscape Engineering, Slovak University of Agriculture, Tr. A. Hlinku 2, 949 76 Nitra, Slovakia; 9 Department of Bioenergetics, Food Analysis and Microbiology, Institute of Food Technology and Nutrition, University of Rzeszow, Cwiklinskiej 1, 35-601 Rzeszow, Poland

**Keywords:** normozoospermia, semen quality, bacterial profiles, cytokines, oxidative stress, antibacterial proteins

## Abstract

This study focused on the identification of bacterial profiles of semen in normozoospermic men and their possible involvement in changes to the sperm structural integrity and functional activity. Furthermore, we studied possible fluctuations of selected cytokines, oxidative markers, and antibacterial proteins as a result of bacterial presence in the ejaculate. Sperm motility was assessed with computer-assisted sperm analysis, while sperm apoptosis, necrosis and acrosome integrity were examined with fluorescent methods. Reactive oxygen species (ROS) generation was quantified via luminometry, sperm DNA fragmentation was evaluated using the TUNEL protocol and chromatin-dispersion test, while the JC-1 assay was applied to evaluate the mitochondrial membrane potential. Cytokine levels were quantified with the biochip assay, whilst selected antibacterial proteins were quantified using the ELISA method. The predominant species identified by the matrix-assisted laser desorption/ionization time-of-flight (MALDI-TOF) mass spectrometry were *Staphylococcus hominis*, *Staphylococcus capitis* and *Micrococcus luteus*. The results revealed that the sperm quality decreased proportionally to the increasing bacterial load and occurrence of conditionally pathogenic bacteria, including *Enterococcus faecalis*, *Staphylococcus aureus* and *Escherichia coli*. Antimicrobial susceptibility tests revealed a substantial resistance of randomly selected bacterial strains to ampicillin, vancomycin, tobramycin, and tetracycline. Furthermore, an increased bacterial quantity in semen was accompanied by elevated levels of pro-inflammatory cytokines, including interleukin-1, interleukin-2, interleukin-6, tumor necrosis factor alpha as well as ROS overproduction and lipid peroxidation of the sperm membranes. Our results suggest that semen quality may be notably affected by the bacterial quantity as well as quality. It seems that bacteriospermia may be associated with inflammatory processes, oxidative stress, sperm structural deterioration, and a subsequent risk for the development of subfertility, even in normozoospermic males.

## 1. Introduction

Bacteriospermia is characterized as the occurrence of bacteria in semen and is clinically recognized when the bacterial count exceeds 1000 colony forming units (CFU)/mL of ejaculate. This condition is often an accompanying phenomenon of acute or chronic bacterial infection of the male urogenital tract that accounts for up to 15% of cases of male sub- or infertility [1]. Different parts of the urogenital system may be compromised by bacterial infestation, including the urethra, testes, epididymides or prostate [2], or the infection may be acquired through sexual intercourse [2,3]. Both G^+^ and G^−^ bacteria may be responsible for bacteriospermia, alongside *Mycoplasma* spp. or *Chlamydia* spp. [1]. While the most prevalent bacteria acknowledged to cause urogenital infections and subsequent bacteriospermia include *Escherichia coli* (*E. coli*), *Staphylococcus aureus* (*S. aureus*), *Ureaplasma urealyticum*, *Chlamydia trachomatis*, *Enterococcus faecalis* (*E. faecalis*), *Mycoplasma* and streptococci [2,4,5], the male reproductive system is not entirely sterile, as it has been already reported that certain bacteria, such as *Staphylococcus capitis* (*S. capitis*), *Staphylococcus epidermidis* (*S. epidermidis*) or *Staphylococcus hominis* (*S. hominis*) are present in otherwise normozoospermic and fertile subjects [6,7,8]. What is more, even in healthy males, semen may become contaminated by microorganisms as it passes through the urogenital system beginning from the testes and moving onward to the penile foreskin [6]. Furthermore, processing and storage of ejaculates are not antiseptic procedures. Additional sources of bacterial contamination may involve collection tools, laboratory glassware or semen diluents. Poor hygiene standards may also be responsible for bacterial infestation of semen [9].

The effects of bacteria on the resulting semen quality are multivariable and have been reported by several studies [3,5,6,7,10,11,12,13,14,15,16,17,18,19,20,21,22,23,24,25,26,27]. Most of the reports agree that bacteriospermia may be accompanied by alterations to the sperm motion and membrane integrity [3,6,10,11], aberrations to the sperm head, mid-piece, or tail, premature acrosome reaction [5,10,12,13], deterioration of the energy metabolism [3,5,7], DNA fragmentation and phosphatidylserine dislocation [10,14,15]. Bacterial adhesion and a subsequent sperm agglutination [5,11,15,16,17] alongside reactive oxygen species (ROS) outbursts and lipid peroxidation (LPO) [18,19,20,21] have also been suggested to be associated with bacteriospermia. Moreover, bacterial infiltration to the male reproductive system has been shown to trigger a local immune reaction that is usually accompanied by the release of cytokines and leukocytospermia [1,3,7,19,22,23,24,25], which have been often associated with a decline of male reproductive performance. Finally, it has been hypothesized that the bacterial metabolism could alter the biochemical or physico-chemical characteristics of the seminal plasma or media used for semen processing, which may endanger the sperm survival under in vivo as well as in vitro conditions [26,27].

Different options are available for the management of bacterial transmission in clinical andrology, including a strict hygiene and sanitation, the use of disinfection agents, and antibacterial substances [28]. Traditionally, antibiotics have become a popular choice to prevent the spread of bacteria, principally because of their cost-effectiveness and availability. Nonetheless, emerging evidence suggests that several bacterial species frequently detected in semen have developed a significant resistance to an array of commonly available antibiotics [29]. Such drug resistance represents a serious threat to public health. Another issue lies in a potential escape of bacteria to the environment and subsequent horizontal transfer of resistance genes to other bacterial species [30]. As such, the use of antibiotics in clinical andrology must be carefully controlled and any potential bacterial resistance patterns should be monitored rigorously [31].

Most currently available studies on human subjects focus on bacteriospermia as a causative agent or an accompanying phenomenon of sub- or infertility [4,5,11,12,21,25]. Nevertheless, it is known that even normozoospermic subjects of good health may produce semen containing bacteria [7,8]. In the era of an increased need for assisted reproductive technologies (ARTs), more emphasis is given to the collection of high-quality semen specimens that will be further processed with media containing antibiotics. As such, besides understanding the etiologies and consequences of bacteriospermia in subfertile subjects, bacteriological analysis of semen in fertile males and their potential resistance to antibiotics should receive equal attention.

Hence, our aim was to characterize the bacterial profiles of ejaculates collected from healthy normozoospermic males, and to study a possible impact of their presence in semen on changes in the sperm quality. Furthermore, we focused on describing any possible fluctuations of a wide array of inflammatory molecules that may play a role in the immune response to bacteriospermia, as well as selected proteins that have been suggested to contribute to the antibacterial protection of male gametes.

## 2. Results

Traditional semen quality characteristics for all studied groups are summarized in Table 1. No significant differences in semen volume or sperm concentration were found among the pre-established groups. In the groups characterized by a high sperm motility (HM; motility > 60%) and acceptable sperm motility (AM; motility > 40%), the leukocyte concentration was significantly higher when compared to the group exhibiting excellent sperm motility (EM; motility > 80%) (*p* < 0.01 in case of HM and *p* < 0.001 for the AM groups, respectively). The motility (MOT) loss in the AM group was accompanied by a significantly increased percentage of Annexin V (AV)—as well as propidium iodide (PI)—positive cells in comparison to the EM (*p* < 0.01) as well as the HM (*p* < 0.05) group. While no significant differences were recorded among the EM and HM group in case of the acrosome integrity or DNA fragmentation indices, a significant acrosome deterioration (*p* < 0.05), sperm chromatin disintegration (*p* < 0.05) and DNA fragmentation (*p* < 0.01) were observed in semen specimens included in the AM group (in comparison to EM).

Selected markers of seminal oxidative balance are provided by Table 2. The lowest ROS production was observed in the EM group which was significantly different from the HM (*p* < 0.05) as well as the AM group (*p* < 0.01). Inversely, total antioxidant capacity (TAC) was significantly lower in comparison with the EM (*p* < 0.01) as well as the HM (*p* < 0.05) group. Proportionately to the highest ROS levels, the highest LPO was detected in the AM group, which was significantly different when compared to the EM (*p* < 0.01) and HM (*p* < 0.05) groups.

The biochip analysis revealed fluctuations in the levels of selected cytokines and growth factors among the pre-established groups (Table 3). Significantly increased concentrations of interleukin-1 alpha (IL-1 α), interleukin-1 beta (IL-1 β) and interleukin-2 (IL-2) were detected in the AM group particularly in comparison with the EM group (*p* < 0.05 with respect to IL-1 β; *p* < 0.01 for IL-1 α; *p* < 0.001 in case of IL-2). While there were no significant differences in the levels of interleukin-4 (IL-4) among the studied groups, the highest concentration of interleukin-6 (IL-6) was recorded in the AM group, which was significantly different in comparison to the EM (*p* < 0.01) as well as the HM group (*p* < 0.05). Similarly, significantly higher interleukin-6 (IL-6), interleukin-8 and interleukin-10 (IL-10) levels were observed in the AM group when compared to the EM group (*p*< 0.01 for IL-6; *p* < 0.05 with respect to IL-8 and IL-10).

Vascular endothelial growth factor (VEGF) levels were unchanged among the studied groups while the concentration of interferon gamma (IFN-γ) was significantly higher in both the HM as well as the AM group when compared to the EM group (*p* < 0.01). The AM group presented with the highest tumor necrosis factor alpha (TNF-α) concentration, which was significant in comparison to both the EM and HM group (*p* < 0.001). In the meantime, no differences in the levels of monocyte chemoattractant protein-1 (MCP-1) and epidermal growth factor (EGF) were found among the pre-established groups.

The sample distribution analysis revealed differences in the levels of proteins that have been suggested to play a role in the protection of semen against bacterial infestation (Table 4). A significantly lower activity of lysozyme was detected in the HM (*p* < 0.05) as well as the AM group (*p* < 0.001) when compared to the EM group. Inversely, the lowest concentration of lactoferrin were recorded in the AM group, which were significant in comparison with the EM group (*p* < 0.01). In the meantime, no differences were found in the levels of phospholipase A2 (PLPA2) among the studied groups.

**Figure 1:**
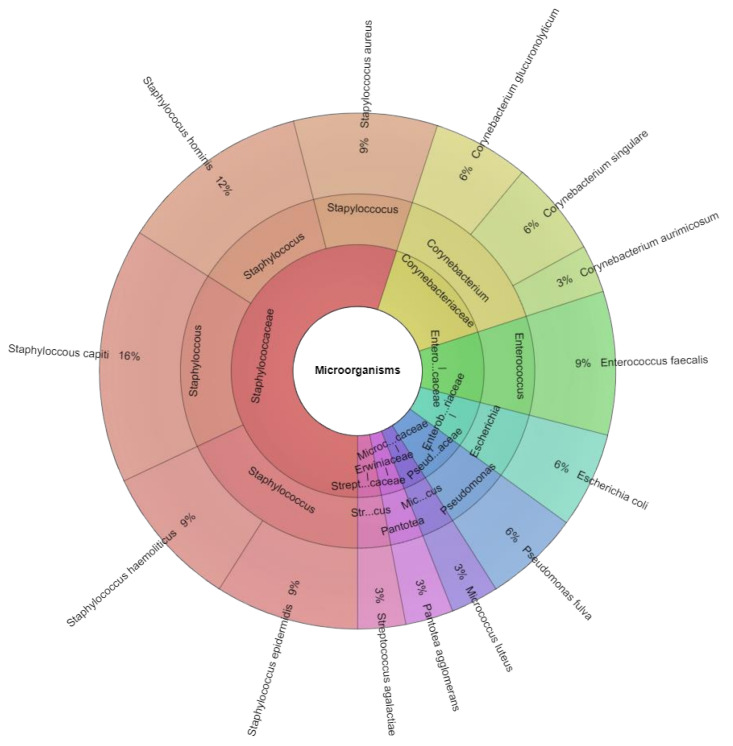
Krona chart of the bacteria identified by MALDI-TOF MS, recovered from human semen (innermost ring: family, middle ring: genus, outermost ring: species).

Matrix assisted laser desorption/ionization time-of-flight (MALDI-TOF) mass spectrometry identified 8 families, 8 genera, and 14 bacterial species in human semen specimes (Figure 1): *Corynebacterium aurimocus* (*C. aurimocus*), *Corynebacterium glucuronolyticum* (*C. glucuronolyticum*), *Corynebacterium singulare* (*C. singulare*), *E. faecalis*, *E. coli*, *Micrococcus luteus* (*M. luteus*), *Pantotea agglomerans* (*P. agglomerans*), *Pseudomonas fulva* (*P. fulva*), *S. aureus*, *S. capitis*, *S. epidermidis*, *Staphyloccoccus haemolyticus* (*S. haemoliticus*), *S. hominis* and *Streptococcus agalactiae* (*S. agalactiae*) (Figure 1).

The highest bacterial load (Table 5) was recorded in the AM group. While no significant differences were observed between the EM and HM groups, a significantly lower number of bacterial colonies was detected in the AM group in comparison to the EM (*p* < 0.001) as well as the HM group (*p* < 0.05). A significant rise in the CFU of coliform bacteria was recorded in the HM (*p* < 0.05) as well as the AM group (*p* < 0.01) when compared to the EM group, which did not contain any coliform bacteria. Semen specimens distributed in the EM group were contaminated with species representing primarily the *Staphylococcus* genus. Additionally, *C. singulare*, *M. luteus* and *P. agglomerans* were identified in the EM group. Staphylococci, corynebacteria, *M. luteus*, *P. agglomerans* and *S. agalactiae* were detected in the HM group; staphylococci, corynebacteria, *E. faecalis*, *S. agalactiae*, *P. agglomerans*, *P. fulva*, *E. coli* and *M. luteus* were found in ejaculates belonging to the AM group.

Randomly selected bacterial isolates were subjected to antimicrobial resistance assessment (supplementary Table S1) against ampicillin (AMP), ceftazidine (CAZ), chloramphenicol (C), imipenem (IMP), linezolid (LZD), norfloxacin (NOR), tetracycline (TE), ticarcillin (TIC), tigecycline (TGC), tobramycin (TOB), and vankomycin (VA). Subsequent inhibition zones were assessed according to the guidelines established by EUCAST (European Committee on Antimicrobial Susceptibility Testing). It was revealed that over 35% of *E. faecalis* isolates were resistant to AMP, while 2 *S*. *aureus* isolates were resistant to TOB. In the case of *S*. *haemolyticus*, 75% of the isolates were resistant to TOB, while another 75% exhibited resistance to TE. Tobramycin revealed to be ineffective against 70% of *S.* *hominis* and 32% of *S*. *capitis* isolates. In the meantime, 68% of *S*. *epidermidis* revealed to be resistant against TE.

**Figure 2:**
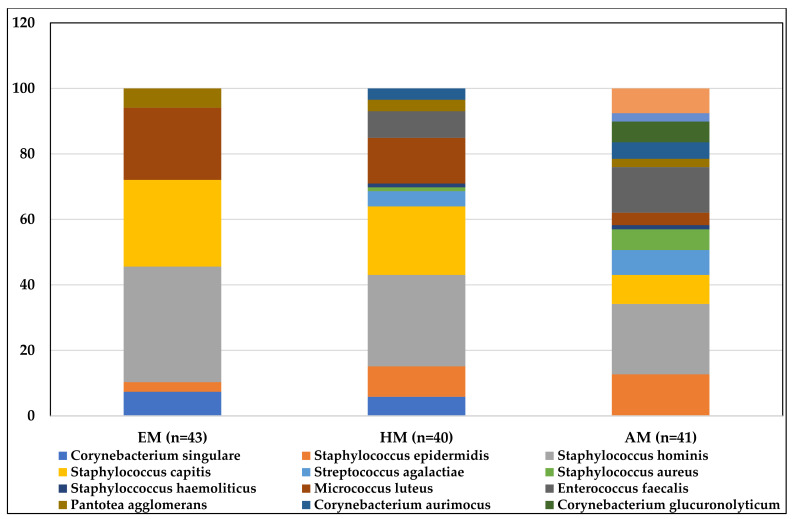
Graphical representation of bacterial species identified in the studied groups and their prevalence. EM—excellent motility group, GM—good motility group, AM—acceptable motility group.

A total of 14 different bacterial species were identified in the collected specimens. The most abundant species in all three groups were *S. hominis*, *S. capitis* and *M. luteus* (Figure 2). The bacterial profile of the AM group was the most diverse, when compared to the EM and HM group.

The biodiversity calculation (Table 6) revealed that the highest bacterial richness was found in the HM group. Dominance index values were low in all pre-established groups, indicating a relatively small domination of a single species in the studied specimens. At the same time, similar values of the Shannon diversity amongst the pre-established groups may have been impacted by the low abundance and quantity of bacteria that were identified in the ejaculates.

## 3. Discussion

Different etiologies are nowadays acknowledged to contribute to a declined male reproductive performance, out of which bacteriospermia has received increased interest from the scientific and clinical community. In order to minimize the loss of sperm vitality and fertilization ability, and to prevent any possible disease transmission to the female, readily available data on the bacterial profiles of semen in health and disease may be paramount for further semen handling in clinical andrology and ARTs.

As mentioned earlier, the presence of bacteria in human ejaculates has been primarily studied in sub- or infertile subjects [2,3,5,12,15,22,32], and it is estimated that depending on the collection procedure and bacteriological analysis, 15–70% samples present with bacteriospermia [7]. Similar to our study, Fraczek et al. [7] strived to describe the microflora of specimens obtained from sexually active men with a normal semen quality. With the help of a complex bacterial screening approach, using traditional techniques and selective media, the study revealed that correspondingly to our results, even ejaculates from normozoospermic males contained a significant number of bacteria. Most of our samples tested positive for G^+^ commensal staphylococci and streptococci, which agrees with Voroshilina et al. [8] and Jedrzejczak et al. [31]. Nevertheless, it must be noted that *E. coli*, *E. faecalis* and *S. haemolyticus*, considered to be conditionally pathogenic bacteria, were detected in our semen specimens, complementing earlier reports by Hou et al. [32] and Fraczek et al. [7], with the former observing no significant differences in the bacterial profiles between healthy semen donors and infertile patients. On the other hand, Fraczek et al. [7] identified anaerobic bacteria such as *Propionibacterium* and *Bacteroides* as well as *Mycoplasma* or *Ureoplasma* in normozoospermic samples, pointing out to the fact that although being a more time- and energy-consuming technique, selective agars still play an important role in clinical bacteriology.

Despite the presence of bacteria in more than 85% of samples included in this study, two factors seem to play a role in potentially detrimental effects of bacteriospermia on male gametes: (a) overall quantity of bacteria present in semen (bacterial load), and (b) bacterial diversity. According to previous studies, the severity of male reproductive dysfunction is generally proportional to the increasing bacterial load in ejaculates [7,32,33,34]. Furthermore, it was revealed that certain bacterial genera may be significantly enriched or depleted in different sperm quality groups [3]. Similar to our bacteriological analysis of the highest quality semen samples, it was previously reported that normozoospermic specimens may carry normal commensals from the male urogenital microflora [7,8,33] or even lactobacilli that may be beneficial for the sperm vitality [3,7,35,36]. Inversely, the microflora of ejaculates that presented with acceptable sperm quality and/or leukocytospermia in this study were richer and included well-known pathogens, such as *E. coli*, *E. faecalis*, *S. aureus* or *S. haemolyticus*, complementing earlier observations by Fraczek et al. [7]. Taking our bacteriological data together, we may agree with Fraczek and Kurpisz [3] suggesting the potential spermatotoxicity of bacteria, since it seems that there is a fine line between a simple contamination of the male reproductive system and a silent bacterial infection.

Another outcome of this study that needs to be taken into consideration, was an increased number of bacterial isolates resistant to an array of antibiotics used for the antimicrobial susceptibility test, including ampicillin, tobramycin, tetracyclin and ticarcillin. According to Al-Jebouri et al. [34], rifampcin, clindamycin or vancomycin were not effective against G^+^ bacteria found in the seminal fluid from Iraqui infertile men. Moreover, G^2212^ bacteria were only moderately sensitive to moxifloxacin, norfloxacin, cefotaxime and ceftriaxone, and were resistant to ceftazidime and cefuroxime. While Isaiah et al. [37] observed that 59% of the bacteria retrieved from the semen of Nigerian males were resistant to oxacillin, Kastrop et al. [38] reported that 90% of the bacterial contaminants of IVF culture dishes were resistant to at least one of the antibiotics commonly used in ARTs. Data on the evolution of bacterial resistance in clinical andrology are sparse; however, our experimental outcomes may reflect a recently published paper observing increased resistance patterns of staphylococci and a rising incidence of methicillin-resistant *S. aureus* in andrology clinics [39]. As such, we may emphasize the necessity of performing screenings of bacterial resistance in ejaculates more frequently in order to re-evaluate the efficiency of antibiotic supplements in semen diluents.

Adverse effects of a bacterial infection on the spermiogram were previously studied in relation to certain bacterial species and/or leukocytes in patients with sub- or infertility [5,6,22,24,40,41]. In this study, however, an increased bacterial load was accompanied by a decline of traditional sperm quality parameters, such as the motility, membrane integrity or mitochondrial activity. These results agree with previous in vivo as well as in vitro reports on human spermatozoa [5,7,10,33,42], indicating changes in the motion behavior as well as in the architecture of male gametes exposed to selected pathological or conditionally pathological bacterial strains, independent of the presence or absence of leukocytes.

A possible involvement of bacteria in the promotion of cell death as observed by an increased incidence of AV-, PI and TUNEL-positive spermatozoa correspondingly to a greater bacterial load and diversity in semen has been suggested earlier by a number of reports observing increased expression patterns of early and/or late apoptotic markers in spermatozoa exposed to pathogenic or conditionally pathogenic bacterial strains [42,43]. Additional correlation studies on patients with bacterial infection have emphasized on an increased incidence of ultrastructural morphological changes typical for cell death [5,14,44]. Moreover, a complete sperm apoptosis and necrosis in spermatozoa from normozoospermic subjects was induced only by a simple contact with bacterial agents without inflammatory processes as revealed by Fraczek et al. [7,42]. Our data agree with this observation, as we recorded a simultaneously decreased mitochondrial membrane potential accompanied by an elevated phosphatidylserine exposure and DNA breakage in spermatozoa, particularly in the presence of typical uropathogenic bacteria. Moreover, inflammation may be involved in the sperm cell death through the cytokine network. It has been speculated that IL-1 β, IL-6, IL-8, or IL-18 could activate the molecular chain of events, leading to the disintegration of the sperm DNA molecule, and a subsequent apoptosis [45]. This hypothesis could be applied to our data as well, since a rise in IL-1 β, IL-6, and particularly IL-8 was observed in the semen samples of the lowest quality that were characterized by the highest pro-apoptotic positivity.

An inherent response to infection lies in the infiltration of leukocytes to the source of inflammation. As postulated by Fraczek and Kurpisz [3], leukocytes are paramount in the surveillance and phagocytosis of abnormal and/or dead spermatozoa; nevertheless, their inappropriate activation ignited by a tight adherence to male gametes leads to phagocytosis, even of viable and morphologically normal spermatozoa [1,3,46,47]. While the negative impact of leukocytospermia on conventional sperm parameters has been previously established in patients suffering from urogenital infections [2,48,49,50], our results emphasize on the fact that the presence of particularly coliform bacteria in semen may be accompanied by a rise of leukocyte levels with a subsequent damage particularly to the sperm membranous structures, even in normozoospermic men. This is in agreement with Fraczek et al. [7], according to who bacteria alongside with leukocytes compromised the lipid symmetry of the sperm membranes in healthy young males, leading to a distortion in the plasma membrane integrity. At this point, however, we must acknowledge that the subjects included in our study were defined as “healthy” simply relying on a subjective perception of the participants, which was not verified by a clinical examination. As such, we cannot rule out that the presence of an elevated bacterial load and leukocyte levels particularly in the AM group may be indicative of a “hidden” infection that should be assessed by a medical professional.

A concomitant mechanism of active immune response lies in the secretion of an array of cytokines, which may inflict damage to male reproductive cells. As emphasized by Fraczek and Kurpisz [3], these biomolecules act within a network, which makes it difficult to assess the spermatotoxicity of just one cytokine. Hence, it seems plausible to hypothesize that the toxicity of one immunomolecule can be modulated in the presence of other components of the immune system. Besides acting as prooxidants primarily through LPO of the sperm membranes [51], it has been hypothesized that cytokines participate in the induction of cell death. Among different pro-inflammatory cytokines analyzed in this study, TNF-α, a predominant cytokine released during inflammation and/or infection, is most often believed to act as an inducer of sperm phosphatidylserine translocation or DNA fragmentation [52,53]. Within the large family of proinflammatory interleukins, IL-1, IL-6 and IL-8 also seem to play an important role in mediating inflammation-inflicted damage to male gametes which agrees with our observations on their increasing levels proportionally to a declining semen quality in the pre-established groups. Accordingly, their increased levels as a response to bacterial overload in semen have been correlated with a decreased sperm quality [54,55]. Similar to TNF-α, interleukins have been previously interconnected with oxidative stress [44,51] and a subsequent decrease in the sperm motion accompanied by an elevated risk for DNA damage [10,45], all of which was reflected by our results as well.

Inflammation within the male genital tract is inherently associated with oxidative stress [3,7]. In this study, we analyzed global ROS production as well as the total antioxidant capacity of semen in order to estimate the overall oxidative balance of the specimens alongside the extent of sperm LPO using the fluorescent BODIPY dye. The intensity of ROS production by bacterial action by and large depends on a set of factors, such as the bacterial quantity, diversity as well as the type of contaminating bacterial strains [3]. The aerobic metabolism of spermatozoa, aerobic as well as facultative anaerobic bacteria predestines them to release ROS as their metabolic by-products. Even anaerobes are equipped with low-potential electron-transfer pathways, enabling them to produce reactive intermediates [56]. Free radicals have been reported to be released by a variety of potentially uropathogenic bacteria, such as *S. aureus* [57], *Bacteroides ureolyticus* [18] and *E. faecalis* [58], additional concentrations of which may contribute to the progression of oxidative damage to spermatozoa. Besides ROS overproduction accompanied by a significant depletion of the antioxidant mechanisms, our data reveal a notable rise in the levels of BODIPY positivity in semen samples, presenting with the highest bacterial load. Sperm membranes are predominantly assembled of polyunsaturated fatty acids (PUFAs), which are highly susceptible to oxidative overload [51]. Excessive amounts of ROS may attack the double bonds present in PUFAs during the process of LPO, which will have a substantial impact on the semipermeable characteristics of the membrane. Our findings may furthermore support the hypothesis that apoptosis could act as an important pathway, leading to ROS-inflicted sperm DNA damage [7]. In this sense, an increased proportion of dead spermatozoa with disintegrated DNA might be a consequence of apoptotic cell death, which has been already observed in the case of male infertility caused by urogenital infections [45,53,59].

An important line of defense against bacterial colonization is represented by innate proteins and enzymes that play diverse roles in the immune activation and protection of spermatozoa against bacteria before entering the female reproductive tract [60,61]. Similar to our results, lysozyme activity was previously reported to be higher in specimens with a high sperm motility and swimming velocity [60]. Inversely, lactoferrin and PLPA2 levels increased proportionately to the bacterial load and occurrence of potentially pathogenic bacteria [60,61], indicating that the synthesis of both proteins may be triggered by an increased presence of pathogens and a subsequent immune response. Nevertheless, data on their bactericidal effects are very sparse, and further elucidation of their behavior during bacteriospermia is needed.

Finally, although several measures were applied prior to and/or during semen collection and handling to minimize the risks for any extrinsic bacterial contamination of the samples, an important limitation of our study lies in numerous “hidden” sources that could potentially contribute to an elevated bacterial load in the ejaculates. As such, data on control samples from the semen donors’ hands, foreskin, urethra, or lab equipment could provide more insight to the origin of the bacteria in the specimens.

## 4. Materials and Methods

### 4.1. Sample Collection and Processing

Semen specimens were collected from 135 healthy volunteers (between 20 and 37 years of age). The inclusion criteria were as follows: (1) normal semen quality parameters according to the World Health Organization (WHO) guidelines [62]; (2) no current or previous urogenital infection; (3) no history of reproductive disease. All donors signed informed consent. All procedures were carried out in accordance with the 1964 Helsinki Declaration and its later amendments or comparable ethical standards. The subjects were asked to urinate, wash their hands and genitalia with soap and dry them using disposable paper towels prior to sample collection. All samples were obtained by masturbation following 2–3 days of abstinence and allowed to liquefy for 30 min at 37 °C in sterile containers. Eleven subjects were excluded for not accomplishing the criteria set by WHO [62].

For a comparative analysis, we chose to divide the samples into groups based on the sperm motility, which to this date remains the most common parameter to assess the quality of semen [63]. The resulting subgroups were determined as follows: samples presenting with an excellent sperm motility (EM; MOT > 80%); samples with high sperm motility (HM; MOT > 60%) and samples exhibiting acceptable sperm motility (AM; MOT > 40%).

Following liquefaction, an aliquot of each specimen was transferred to an Eppendorf tube and stored at −80 °C for bacteriological analysis. A second semen aliquot was centrifuged at 300× *g* and 20 °C for 10 min. The obtained seminal plasma was stored at −80 °C for the biochip and ELISA assays. The final aliquot of native semen was used for the assessment of conventional as well as non-conventional sperm characteristics.

### 4.2. Assessment of Semen Quality

Sperm motility was assessed with the HTM TOX IVOS II. Computer-assisted semen analysis (CASA) system (version 14.0; Hamilton-Thorne Biosciences, Beverly, MA, USA). A total of 100 μL of each semen specimen was transferred into a clean microcentrifuge tube and mixed with 50 μL of pre-warmed phosphate buffered saline (Dulbecco’s PBS, without calcium chloride and magnesium chloride; Sigma-Aldrich, St. Louis, MO, USA). Subsequently, 150 μL of the diluted semen sample was mixed with 50 of the IDENT stain (final concentration of 10 μg/mL; Hamilton-Thorne Biosciences, Beverly, MA, USA) and incubated at 37 °C for 10 min. Then, 10 μL of the mixture was placed into the analyzer and at least 300 cells were assessed under fluorescent illumination [64,65].

Commercially available Annexin-V-FLUOS kit (Roche Applied Science, Basel, Switzerland) was used to evaluate the sperm membrane stability. A dual staining protocol with Annexin-V (AV) and propidium iodide (PI) enabled to distinguish cells in the early stage of apoptosis (AV^+^/PI^2212^), necrotic or late-stage apoptotic cells (AV^+^/PI^+^) or living cells (AV^2212^/PI^2212^) [65]. Sperm suspensions adjusted with pre-warmed PBS (Sigma-Aldrich, St. Louis, MO, USA) to 10^6^ cells were pre-treated with 50 μL of incubation buffer, 1 μL AV and 1 μL PI. Following incubation at 37 °C for 30 min, the samples were stained with 10 μL DAPI (4′,6-diamidino-2-phenylindole; Sigma-Aldrich, St. Louis, MO, USA; 1 μM in PBS), and at least 300 cells were observed under an epifluorescence microscope with a 40× magnification objective (Leica Microsystems, Wetzlar, Germany) [65,66]. The data are expressed as % apoptotic cells and % necrotic cells, respectively.

For the acrosome integrity, 10^6^ spermatozoa were stained with 100 μL PNA (peanut agglutinin, FITC conjugate; Sigma-Aldrich, St. Louis, MO, USA; 10 μM in PBS) and 10 μL DAPI. Following incubation at 37 °C for 30 min, at least 300 cells were counted under an epifluorescence microscope (40×) in each sample. Spermatozoa negative for the PNA stain were classified as acrosome-intact (%) [67].

Mitochondrial activity was examined with the Mitochondrial Membrane Potential Assay Kit (Cayman Chemical, Ann Arbor, MI, USA). One million spermatozoa were adjusted to 100 μL with PBS and stained with 5 μL JC-1 working solution. Following incubation at 37 °C for 20 min, the cells were centrifuged at 150× *g* and 20 °C for 10 min, washed twice with PBS, transferred to a black 96-well plate and JC-1 monomers and polymers were quantified with a combined spectro-fluoro-luminometer (Glomax Multi+; Promega Corporation, Madison, WI, USA). Mitochondrial membrane potential (ΔΨm) is expressed as the ratio of JC-1 complexes to JC-1 monomers (red/green ratio) [65].

Sperm chromatin integrity was assessed with the Halosperm**^®^** commercial kit (Halotech DNA, Madrid, Spain). Semen samples (20 μL) were mixed with low-melting point agarose, placed on a microscopic slide pre-coated with agarose, and cooled down to 4 °C. Once the agarose was solidified, the slides were exposed to an acid solution (7 min), a lysis solution (20 min) and subsequently washed with distilled water (5 min). Finally, the slides were dehydrated in 70% and 100% ethanol for 2 min each and air-dried. Following staining with Sybr-Green (final concentration of 2 μg/mL; Sigma Aldrich, St. Louis, MO, USA) at least 300 spermatozoa were scored under an epifluorescence microscope (40×) [68]. The proportion of spermatozoa with damaged chromatin is expressed in %.

Sperm DNA fragmentation was assessed with the Apo-DIRECT™ kit (Thermo Fisher Scientific, Waltham, MA, USA). The specimens were adjusted with pre-warmed PBS to 2.5 × 10^6^ sperm, centrifuged at 300× *g*, 20 °C for 10 min and washed twice in PBS (Sigma Aldrich, St. Louis, MO, USA). Following cell fixation in 4% (*w*/*v*) paraformaldehyde (Sigma Aldrich, St. Louis, MO, USA) for 15 min, and centrifugation (at 300× *g*, 20 °C, 10 min), spermatozoa were permeabilized in 70% (*v*/*v*) ice-cold ethanol and kept at **−**20 °C. For the assay, the cells were centrifuged at 300× *g* and 20 °C for 10 min and washed twice with a wash buffer, stained with 50 µL of the staining solution comprising a reaction buffer, TdT enzyme, FITC-dUTP and distilled water, and incubated for 60 min at 37 °C. At the end of the incubation, 1 mL of rinse buffer was added, and the samples were centrifuged twice. Finally, 0.5 mL of propidium iodide (PI)/RNase solution was added, the specimens were transferred to a black 96-well plate and assessed with the Glomax Multi^+^ combined spectro-fluoro-luminometer. TUNEL-positivity is expressed in % [69].

The presence of leukocytes was assessed with the Endtz test. A total of 20 µL of liquefied specimens was treated with 40 µL of Endtz solution containing 96% ethanol (Centralchem, Bratislava, Slovakia), benzidine (Sigma-Aldrich, St. Louis, MO, USA), sterile water, and 3% hydrogen peroxide (H_2_O_2_; Sigma-Aldrich, St. Louis, MO, USA). Following incubation (20 °C, 5 min), stained round cells were counted under a bright-field microscope (Nikon ECLIPSE E100, Tokyo, Japan; 1000×). The results are expressed as × 10^6^ leukocytes/mL [70].

### 4.3. Oxidative Profile

The chemiluminescent technique to assess the extent of ROS production in the samples relies on a direct quantification of intracellular as well as extracellular ROS with the help of luminol [71]. Semen specimens (100 μL) were transferred to a 96-well plate and exposed to 2.5 μL of 5 mM luminol (Sigma-Aldrich, St. Louis, MO, USA). Negative controls comprised 100 μL PBS and 2.5 μL luminol, while positive controls included 100 μL PBS, 12.5 μL H_2_O_2_ (33%; Sigma-Aldrich, St. Louis, MO, USA) and 2.5 μL luminol. The light signal emitted from the interaction of ROS and luminol was monitored with the Glomax Multi^+^ combined spectro-fluoro-luminometer (Promega Corporation, Madison, WI, USA). The experimental results are expressed in relative light units per second per million sperm (RLU/s/10^6^ sperm) [65].

Total antioxidant capacity of semen was assessed with a chemiluminescent assay introduced by Muller et al. [72], which takes advantage of a signal reagent comprised of luminol, horseradish peroxidase (HRP; Sigma-Aldrich; St. Louis, MO, USA), 4-iodophenol (Sigma-Aldrich; St. Louis, MO, USA), and H_2_O_2_. Chemiluminescence was monitored during 10 consecutive cycles of 1 min with with the Glomax Multi^+^ combined spectro-fluoro-luminometer (Promega Corporation, Madison, WI, USA). Calculation of the results was carried out using a Trolox (5–100 μmol/L; 6-hydroxy-2,5,7,8-tetramethylchroman-2-carboxylic acid; Sigma-Aldrich; St. Louis, MO, USA) standard curve. The results are expressed as μmol Trolox Eq./L.

For the evaluation of lipid peroxidation, BODIPY**^®^** 581/591 C11 (5 µM, Thermo Fisher Scientific, Waltham, MA, USA) was added to 2 × 10^6^ spermatozoa and incubated at 37 °C for 30 min. The cells were washed twice with PBS and at least 300 cells were evaluated under an epifluorescence microscope with a 40× magnification objective (Leica Microsystems, Wetzlar, Germany). The localization of the oxidized probe emission was assessed at a wavelength range of 495–545 nm (green fluorescence), whereas the intact probe emission was selected at the wavelength range of 580–620 nm (red fluorescence) [73]. BODIPY-C11^+^ cells are expressed in %.

### 4.4. Biochip Assay

Selected cytokines and growth factors were measured using the Randox Evidence Investigator and the Cytokine & Growth Factors Array (Randox Laboratories, Crumlin, UK). This sandwich chemiluminescent immunoassay contains a variety of discrete test regions of immobilized antibodies specific to IL-1α, IL-1β, Il-2, IL-4, IL-6, IL-8, IL-10, IFN γ, TNF α, VEGF, MCP-1 and EGF. The light signal generated from each test region on the Biochip with antibodies labeled with HRP is detected using a device camera and compared to a stored calibration curve.

Seminal plasma specimens were diluted with an assay buffer and applied to a biochip, which was incubated at 37 °C and shaken at 370 RPM and 20 °C for 60 min. After washing, the conjugate was added and again incubated (37 °C) and shaken (370 RPM, 20 °C, 60 min). After washing, 250 mL of a mix of luminol and peroxide (1:1) was added and incubated for 2 min (20 °C). Finally, the carrier was processed with the help of the Investigator System with a digital imaging technology [74].

### 4.5. ELISA

Levels of lysozyme, lactoferrin and phospholipase A were quantified using specific commercially available ELISA kits (#ab108880, #ab200015, and #ab133089, respectively; Abcam, Cambridge, UK). All assays employed a double-sandwich ELISA methodology and were carried out on 96-well plates with the help of the Glomax plate spectrophotometer (Promega, Madison, WI, USA) at 450 nm.

### 4.6. Bacteriological Analysis

For the identification of the bacterial species in semen, 100 µL of each specimen was inoculated on selected sterile agars (Gassner agar, blood agar base no. 2, MacConkey agar; trypticase soy agar; Merck, Darmstadt, Germany) and incubated under aerobic conditions at 36 ± 2 °C for 24 h. The resulting bacterial colonies were counted and re-inoculated to fresh agars to obtain pure cultures, which were incubated again under aerobic conditions at 37 ± 1 °C for 24 h [75].

Individual bacterial colonies were identified with the matrix assisted laser desorption/ionization time-of-flight (MALDI-TOF) Biotyper mass spectrometry (Brucker Daltonics, Bremen, Germany). Purified cultures were re-suspended in 300 μL distilled water. Afterward, 900 μL 99.8% ethanol (Sigma-Aldrich, St. Louis, MO, USA) was added and the mixture was centrifuged (920× *g*, 20 °C, 2 min). The resulting dry pellet was mixed thoroughly with 30 μL of acetonitrile (Sigma-Aldrich, St. Louis, MO, USA), 30 μL 70% formic acid (Sigma-Aldrich, St. Louis, MO, USA), and centrifuged at 1096× *g*, 20 °C for 2 min. Subsequently, 1 μL of the supernatant was transferred to the MALDI identification plate, dried and covered with a working solution of MALDI matrix containing ultrapure water, acetonitrile, trifluoroacetic acid and cinnamic acid (Sigma-Aldrich, St. Louis, MO, USA). Bacterial identification was carried out with the Microflex LT instrument equipped with the flexControl software (version 3.4, Singapore). The obtained spectra were linked with the MALDI Biotyper Bruker Taxonomy database (Bruker Daltonics, Bremen, Germany) [75].

Randomly selected bacterial isolates were furthermore assessed for antibiotic resistance. The antimicrobial susceptibility test was carried out using the disc diffusion method against (10 mg) ampicillin (AMP), ceftazidine (CAZ), chloramphenicol (C), imipenem (IMP), linezolid (LZD), norfloxacin (NOR), tetracycline (TE), ticarcillin (TIC), tigecycline (TGC), tobramycin (TOB), and vankomycin (VA) as previously described by Kačániová et al. [75].

### 4.7. Biodiversity Calculation

Differences among the analyzed groups were calculated with the Astasta calculator and one-way ANOVA. The overall number of species obtained from the pre-established groups of samples was defined as species richness. Standard α-, β-, and γ-diversity parameters were assessed with the BPMSG diversity calculator. Shannon alpha entropy was calculated according to the formula:

Hα =−w1∑Ni = 1pi1ln pi1 + (−w2∑Ni) = 1pi2ln pi2 +⋯+ wK∑N1piKln piK

where pij is the relative abundance (frequency, priority, share) of class i and sample j and wi statistical weights of samples; ∑Ki = 1wi = 1. The Berger–Parker index was gathered following the formula d = max(pi) to describe real unbalanced groups [76].

### 4.8. Statistics

GraphPad Prism (version 9.0 for Mac; GraphPad Software Incorporated, La Jolla, CA, USA) was used for the statistical analysis. The results are expressed as median (25th; 75th percentile). Differences between the established groups were analyzed by one-way ANOVA and Tukey multiple comparison test. Statistical significance was set at * *p* < 0.05; ** *p* < 0.01; *** *p* < 0.001.

## 5. Conclusions

In conclusion, our study suggests that bacteriospermia could be present, even in semen specimens of normozoospermic men. Our experiments have revealed that the sperm quality may be equally affected by the bacterial quantity as well as diversity. Ejaculates with a higher bacterial load contained a significantly higher proportion of apoptotic male gametes with disrupted membranes and DNA, which could have been translated into the loss of sperm motility and vitality. Furthermore, our data indicate an important role of oxidative stress and cytokine network in the promotion in the bacteria-inflicted sperm damage. An important aspect of this study may lie in the involvement of antibacterial proteins in the maintenance of the sperm survival during bacterial infestation of semen. Finally, we may emphasize the importance of microbiological screenings of ejaculates in clinical practice, including a regular assessment of bacterial resistance patterns to antibiotics.

## Figures and Tables

**Table 1:** Semen quality of the studied groups.

	Excellent Motility (n = 43)	High Motility (n = 40)	Acceptable Motility (n = 41)
Volume [mL]	3.30 (2.80; 3.80)	2.90 (2.20; 4.10)	3.25 (1.90; 3.50)
Concentration [×10^6^/mL]	77.07 (56.90; 81.30)	73.16 (49.90; 81.80)	74.21 (48.70; 83.13)
Motility [%]	83.00 (80.00; 87.00)	75.00 (65.00; 76.00) *^EM^	50.00 (44.25; 55.75) ***^EM;^ ***^HM^
AV-positive cells [%]	14.79 (13.60;18.36)	19.15 (16.39; 22.55)	24.62 (18.14; 29.04) **^EM;^ *^HM^
PI-positive cells [%]	3.79 (2.95; 4.73)	6.23 (5.32; 10.50)	9.61 (8.89; 10.78) **^EM;^ *^HM^
Mitochondrial membrane potential [green/red ratio]	0.73 (0.44; 0.78)	0.67 (0.40; 0.72)	0.49 (0.34; 0.74) **^EM;^ *^HM^
Acrosome integrity [%]	83.89 (80.58; 85.01)	79.26 (76.44; 82.19)	74.41 (68.31; 78.43) *^EM^
Chromatin-dispersion test [%]	12.42 (10.85; 13.99)	15.21 (12.85; 18.74)	20.99 (19.42; 25.70) *^EM^
TUNEL-positive cells [%]	14.05 (13.25; 17.00)	18.55 (16.00; 20.76)	23.93 (16.02; 25.88) **^EM^
Leukocyte concentration [×10^6^/mL]	0.31 (0.00; 0.80)	2.45 (0.00; 8.00) **^EM^	5.54 (0.40; 10.00) ***^EM;^ **^HM^

Values are represented as median (25th; 75th percentile). * *p* < 0.05; ** *p* < 0.01; *** *p* < 0.001. ^EM^—versus excellent motility group; ^HM^—versus high motility group. AV—annexin V, PI—propidium iodide.

**Table 2:** Oxidative characteristics of semen collected from the studied groups.

	Excellent Motility (n = 43)	High Motility (n = 40)	Acceptable Motility (n = 41)
ROS production [RLU/s/10^6^ sperm]	2.84 (1.07; 4.36)	5.99 (4.68; 7.32) *^EM^	9.70 (6.62; 11.53) **^EM;^ *^HM^
Total antioxidant capacity [eq. μmol Trolox/L]	16.58 (9.91; 19.06)	9.95 (7.51; 12.34) *^EM^	6.40 (2.81; 8.44) ^**EM;^ *^HM^
BODIPY-positive cells [%]	5.28 (2.32; 6.96)	10.21 (8.20; 12.18) *^EM^	15.71 (10.63; 18.72) **^EM;^ *^HM^

Values are represented as median (25th; 75th percentile). * *p* < 0.05; ** *p* < 0.01. ^EM^—versus excellent motility group; ^HM^—versus high motility group.

**Table 3:** Immune profile of semen collected from the studied groups.

	Excellent Motility (n = 43)	High Motility (n = 40)	Acceptable Motility (n = 41)
IL-1 α [pg/mL]	4.30 (1.63; 22.87)	5.05 (2.50; 22.48)	10.44 (2.80; 26.89) **^EM;^ **^HM^
IL-1 β [pg/mL]	8.82 (6.11; 10.94)	11.08 (8.40; 16.76)	13.10 (8.78; 32.83) *^EM^
IL-2 [pg/mL]	0.42 (0.00; 1.00)	2.43 (0.00; 7.56) **^EM^	4.51 (0.00; 8.29) ***^EM;^ **^HM^
IL-4 [pg/mL]	1.72 (1.57; 2.03)	1.95 (1.57; 2.23)	2.03 (1.72; 2.26)
IL-6 [pg/mL]	7.45 (2.72; 12.92)	10.76 (3.92; 15.02)	14.27 (6.55; 26.08) **^EM;^ *^HM^
IL-8 [pg/mL]	715.50 (580.2; 1317)	891.90 (594.60; 1351)	1236.00 (699.20; 1351) *^EM^
IL-10 [pg/mL]	11.38 (10.22; 19.23)	15.54 (14.20; 20.07)	21.08 (13.91; 57.47) *^EM^
VEGF [pg/mL]	3200.00 (2168.00; 3349.00)	3132.00 (2597.00; 3749.00)	2957.00 (2449.00; 3075.00)
IFN-γ [pg/mL]	0.47 (0.00; 0.68)	2.86 (0.00; 3.12) **^EM^	2.99 (0.00; 24.71) **^EM^
TNF-α [pg/mL]	0.15 (0.00; 0.37)	0.26 (0.00; 0.86)	6.56 (0.00; 8.24) ***^EM;^ ***^HM^
MCP-1 [pg/mL]	1414.00 (776.60; 1596.00)	1596.00 (1104.00; 1720.00)	1596.00 (1260.00; 1705.00)
EGF [pg/mL]	1197.00 (1039.00; 1500.00)	1197.00 (1001.00; 1444.00)	1200.00 (1020.00; 1450.00)

Values are represented as median (25th; 75th percentile). * *p* < 0.05; ** *p* < 0.01; *** *p* < 0.001. ^EM^—versus excellent motility group; ^HM^—versus high motility group. IL-1 α—interleukin-1 alpha, IL-1 β—interleukin-1 beta, IL-2—interleukin-2, IL-6—interleukin-6, IL-8—interleukin-8, IL-10—interleukin-10, VEGF—vascular endothelial growth factor, IFN-γ—interferon gamma, TNF-α—tumor necrosis factor alpha, MCP-1—monocyte chemoattractant protein-1, EGF—epidermal growth factor.

**Table 4:** Concentrations of selected antibacterial proteins in semen collected from the studied groups.

	Excellent Motility (n = 43)	High Motility (n = 40)	Acceptable Motility (n = 41)
Lysozyme [U/L]	3.76 (3.45; 4.02)	3.02 (2.55; 3.33) *^EM^	1.97 (1.48; 2.19) ***^EM;^ ***^HM^
Lactoferrin [mg/100 mL]	11.20 (10.00; 12.80)	12.90 (11.55; 13.93)	13.55 (13.15; 14.53) **^EM^
PLPA2 [ng/mL]	0.87 (0.76; 0.90)	0.89 (0.79; 0.88)	0.99 (0.82; 1.19)

Values are represented as median (25th; 75th percentile). * *p* < 0.05; ** *p* < 0.01; *** *p* < 0.001. ^EM^—versus excellent motility group; ^HM^—versus high motility group. PLPA2—phospholipase A2.

**Table 5:** Bacteriological characterization of semen collected from the pre-established groups.

	Excellent Motility (n = 43)	High Motility (n = 40)	Acceptable Motility (n = 41)
Bacterial colonies [log_10_ CFU/mL]	3.84 (3.71; 3.94)	4.19 (3.88; 4.29)	4.34 (4.26; 4.71) ***^EM;^ *^HM^
Coliform bacteria [log_10_ CFU/mL]	0.00 (0.00; 0.00)	1.83 (1.62; 2.28) **^EM^	2.36 (2.23; 2.64) ***^EM^
Number of samples without any detected bacteria	11/43	7/40	0/41
Bacterial species (sample positivity)	*Staphylococcus hominis* (24/43) *Staphylococcus capitis* (18/43) *Micrococcus luteus* (15/43) *Corynebacterium singulare* (5/43) *Pantotea agglomerans* (4/43) *Staphylococcus epidermidis* (2/43)	*Staphylococcus hominis* (24/40) *Staphylococcus capitis* (17/40) *Micrococcus luteus* (12/40) *Staphylococcus epidermidis* (8/40) *Corynebacterium singulare* (5/40) *Enterococcus faecalis* (5/40) *Streptococcus agalactiae* (4/40) *Corynebacterium aurimocus* (3/40) *Pantotea agglomerans* (3/40) *Staphylococcus aureus* (1/40) *Staphyloccoccus haemoliticus* (1/40)	*Staphylococcus hominis* (21/41) *Staphyloccoccus haemolyticus* (18/41) *Enterococcus faecalis* (17/41) *Staphylococcus epidermidis* (16/41) *Escherichia coli* (7/41) *Staphylococcus capitis* (7/41) *Streptococcus agalactiae* (6/41) *Corynebacterium glucuronolyticum* (5/41) *Staphylococcus aureus* (5/41) *Corynebacterium aurimocus* (4/41) *Micrococcus luteus* (3/41) *Pantotea agglomerans* (2/41) *Pseudomonas fulva* (2/41)

Values are represented as median (25th; 75th percentile). * *p* < 0.05; ** *p* < 0.01; *** *p* < 0.001. ^EM^—versus excellent motility group; ^HM^—versus high motility group.

**Table 6:** Bacterial diversity in human semen samples divided into three groups based on their motility.

	Excellent Motility (n = 43)	High Motility (n = 40)	Acceptable Motility (n = 41)
Average population size	7.43	7.81	5.69
Richness (R)	6.00	11.00	13.00
Berger Parker Index/Dominance Index	0.46	0.279	0.229
Shannon α-diversity	1.87	2.15	3.29
β-diversity	0.34	0.16	0.12

## Data Availability

The data presented in this study are available on request from the corresponding author.

## Supplementary Materials

The supporting information can be downloaded at: https://www.mdpi.com/article/10.3390/ijms23158678/s1.
