# Supplementary material for: Possible Implications of Bacteriospermia on the Sperm Quality, Oxidative Characteristics, and Seminal Cytokine Network in Normozoospermic Men
Source: Int J Mol Sci. 2022 Aug 4;23(15):8678. doi: 10.3390/ijms23158678 (PMC9369207; doi:10.3390/ijms23158678)
Supplement: Supplementary file 1 [file ijms-23-08678-s001.zip › ijms-1824460-supplementary.pdf]

Supplementary Table S1. Resistance profiles of randomly selected bacterial isolates recovered from human semen.

Bacterium	Isolate	LZD	AMP	TGC	IMP
E. faecalis	1	S	S	S	S
	2	S	S	S	S
	3	S	S	S	S
	4	S	S	S	S
	5	S	R	S	S
	6	S	S	S	S
	7	S	R	S	S
	8	S	S	S	S
	9	S	R	S	S
	10	S	R	S	R
	11	S	S	S	S
	12	S	S	S	S
	13	S	S	S	S
	14	S	R	S	R

Bacterium	Isolate	TE	C	LZD	VA
S. agalactiae	1	S	S	S	R
	2	R	R	S	S
	3	S	S	S	S
	4	S	S	S	S
	5	R	R	S	S
	6	S	S	S	S

Bacterium	Isolate	TOB	C	TGC	TE
S. haemolyticus	1	R	S	S	R
	2	R	S	S	R
	3	R	S	S	R
	4	S	S	S	R
	5	R	S	S	R
	6	R	S	S	R
	7	R	S	S	R
	8	R	S	S	R
	9	S	S	S	R
	10	R	S	S	S
	11	R	S	S	R
	12	S	S	S	R
	13	S	S	S	R
	14	R	S	S	I
	15	R	S	S	R
	16	R	S	S	S
	17	S	S	S	I
	18	R	S	S	R
	19	R	S	S	R
	20	R	S	S	S

Bacterium	Isolate	TOB	C	TGC	TE
S. capitis	1	S	S	S	R
	2	S	S	S	R
	3	S	S	S	S
	4	S	S	S	S

Bacterium	Isolate	TIC	TOB	C	NOR
E. coli	1	R	S	S	S
	2	R	S	S	S
	3	S	S	S	S

Bacterium	Isolate	TIC	TOB	CAZ	IMP
P. aglomerans	1	R	S	R	S
	2	S	S	S	S
	3	S	S	S	S

Bacterium	Isolate	TOB	C	TGC	TE
S. aureus	1	S	S	S	S
	2	R	S	S	S
	3	S	S	S	R
	4	R	S	S	S

Bacterium	Isolate	TOB	C	TGC	TE
S. epidermidis	1	R	S	S	R
	2	S	S	S	R
	3	S	S	S	R
	4	R	S	S	R
	5	S	S	S	R
	6	R	S	S	I
	7	R	S	S	R
	8	R	S	S	S
	9	R	S	S	S
	10	S	S	S	R
	11	R	S	S	R
	12	S	S	S	R
	13	S	S	S	R
	14	R	S	S	S
	15	R	S	S	S
	16	R	S	S	S
	17	R	S	S	R
	18	S	S	S	R
	19	S	S	S	R

Bacterium	Isolate	TOB	C	TGC	TE
S. hominis	1	S	S	S	R
	2	R	S	S	R
	3	R	S	S	R
	4	R	S	S	I
	5	S	S	S	R
	6	R	S	S	R
	7	R	S	S	I
	8	R	S	S	R
	9	S	S	S	R
	10	S	S	S	R
	11	R	S	S	I
	12	S	S	S	R

5	S	S	S	R
6	R	S	S	I
7	S	S	S	R
8	S	S	S	R
9	S	S	S	R
10	R	S	S	I
11	R	S	S	I
12	S	S	S	R
13	S	S	S	R
14	R	S	S	I
15	S	S	S	S
16	S	S	S	S
17	S	S	S	R
18	R	S	S	I
19	R	S	S	I
20	S	S	S	R
21	S	S	S	R
22	S	S	S	R
23	S	S	S	R
24	R	S	S	I
25	R	S	S	I
26	S	S	S	S
27	S	S	S	S
28	S	S	S	R
29	S	S	S	R
30	R	S	S	I
31	R	S	S	I

	13	S	S	S	R
	14	S	S	S	R
	15	R	S	S	S
	16	R	S	S	S
	17	R	S	S	S
	18	R	S	S	R
	19	R	S	S	R
	20	S	S	S	R
	21	R	S	S	R
	22	R	S	S	R
	23	R	S	S	R
	24	R	S	S	I
	25	R	S	S	R
	26	R	S	S	R
	27	R	S	S	R
	28	S	S	S	R
	29	S	S	S	R
	30	R	S	S	S
	31	R	S	S	S
	32	R	S	S	S
	33	R	S	S	S
	34	R	S	S	S
	35	S	S	S	R
	36	R	S	S	R
	37	R	S	S	I
		**TIC**	**TOB**	**CAZ**	**IMP**
P. fulva	*1*	S	S	S	S

AMP – ampicillin, CAZ – ceftazidine, C – chloramphenicol, IMP – imipenem, LZD – linezolid, NOR – norfloxacin, TE – tetracycline, TIC – ticarcillin, TGC – tigecycline, TOB – tobramycin, VA – vankomycin. S–sensitive, I–intermediate, R–resistant.
